# School environment, socioeconomic status and weight of children in Bloemfontein, South Africa

**DOI:** 10.4102/phcfm.v7i1.751

**Published:** 2015-03-31

**Authors:** Lucia N.M. Meko, Marthinette Slabber-Stretch, Corinna M. Walsh, Salome H. Kruger, Mariette Nel

**Affiliations:** 1 Nutrition and Dietetics, University of the Free State, South Africa; 2 School of Physiology and Nutrition, North-West University, Potchefstroom Campus, South Africa; 3 Biostatistics, Faculty of Health Sciences, University of the Free State, South Africa

## Abstract

**Background:**

The continued existence of undernutrition, associated with a steady increase in the prevalence of overweight and obesity in children and adolescents, necessitates identification of factors contributing to this double burden of disease, in order for effective treatment and prevention programmes to be planned.

**Aim:**

To determine the nutritional status of 13–15-year-old children in Bloemfontein and its association with socioeconomic factors.

**Setting:**

Bloemfontein, Free State Province, South Africa (2006).

**Methods:**

This was a cross-sectional analytical study. Randomly selected children (*n* = 415) completed structured questionnaires on socioeconomic status. The children's weight and height were measured and body mass index-for-age and height-for-age *z*-scores were computed according to World Health Organization growth standards in order to determine the prevalence of underweight, overweight, obesity and stunting. Waist circumference was measured to classify the children as having a high or very high risk for metabolic disease.

**Results:**

Of the 415 children who consented to participate in the study, 14.9% were wasted and 3.4% were severely wasted. Only 6% of the children were overweight/obese. Significantly more boys (23.0%) were wasted than girls (10%) and severe stunting was also significantly higher in boys than in girls (10.3% and 4.2%, respectively). Children whose parents had graduate occupations were significantly more overweight/obese than those with parents working in skilled occupations. Stunting was significantly higher in low (31.4%) and medium (30.4%) socioeconomic groups compared to the high socioeconomic group (18.1%).

**Conclusion:**

A coexistence of underweight and overweight was found and gender and parental occupation were identified as being predictors of nutritional status.

## Abstrait

**Environnement scolaire, Statut socioéconomique et poids des enfants à Bloemfontein, Afrique du Sud.**

**Contexte:**

La permanence de la sous-alimentation, associée à l'augmentation graduelle de l'obésité et du surpoids chez les enfants et les adolescents, nécessite d'identifier les facteurs qui contribuent à ce double fardeau de maladie, afin de mettre en place des programmes de prévention et de traitement.

**Objectif:**

Déterminer l’état nutritionnel des enfants de 13 à 15 ans à Bloemfontein et son rapport avec des facteurs socioéconomiques.

**Cadre:**

Bloemfontein, Province du Free State, Afrique du Sud (2006).

**Méthodes:**

C’était une étude analytique transversale. Des enfants sélectionnés au hasard (*n* = 415) ont rempli des questionnaires structurés sur leur état socioéconomique. On a mesuré le poids et la taille des enfants ainsi que l'indice de masse corporelle pour leur âge et le rapport taille-âge. Les résultats-z ont été saisis selon les normes de croissance de l'Organisation mondiale de la Santé afin de déterminer la fréquence de sous-poids, surpoids, obésité et retard de croissance. Le tour de taille des enfants a été mesuré pour les classer selon leurs risques élevés ou très élevés de maladies métaboliques, respectivement.

**Résultats:**

Parmi les 415 enfants qui ont accepté de participer à l’étude, 14.9% étaient malingres et 3.4% étaient décharnés. Seuls 6% des enfants étaient en surpoids/obèses. Il est significatif que plus de garçons (23.0%) étaient malingres que de filles (10%) et le retard de croissance étaient beaucoup plus élevé chez les garçons que chez les filles (10.3% et 4.2%, respectivement). Les enfants dont les parents étaient diplômés étaient beaucoup plus en surpoids/obèses que ceux des parents peu qualifiés. Le retard de croissance était plus élevé chez les groupes socio-économiquement faibles (31.4%) et moyens (30.4%) que dans le groupe socio-économiquement élevé (18.1%).

**Conclusion:**

On a remarqué la coexistence des enfants en sous-poids et en surpoids et le sexe et la profession des parents étaient des indicateurs de leur état nutritionnel.

## Introduction

The prevalence of obesity amongst children in developing countries is increasing and childhood obesity has become a serious public health challenge of the 21st century. Countries undergoing rapid economic development, such as Brazil, China and South Africa, are experiencing a double burden of both under- and overnutrition.^1,2,3^ Although undernutrition, which is caused by energy and micronutrient deficiencies, shows a decline in many parts of the world, it is still increasing in most parts of Africa.^4^ The prevalence rates of overweight and obesity in African countries, on the other hand, are showing a pattern similar to that of developed countries.^4^ It is estimated that by the year 2015, non-communicable diseases resulting from overnutrition will overtake undernutrition as a leading cause of death in developing countries.^5,6^

Both undernutrition and overnutrition are known to have undesirable health effects. Malnutrition in childhood and adolescence can lead to growth retardation and delays in sexual maturation, amongst others,^7^ whilst overweight and obesity may lead to a number of chronic illnesses such as hyperlipidaemia, hyperinsulinaemia, hypertension and early atherosclerosis emerging in childhood rather than in adulthood.^8,9^

‘South Africa has a complex mix of developed areas in terms of its population and economy. The gap in income distribution between the poor majority and wealthy minority is huge’.^10^ This unequal distribution is reflected by the high prevalence of stunting in black children, especially in rural areas, accompanied by a high prevalence of overweight and obesity in all ethnic groups residing in urban areas.^10^

In developed countries, the prevalence of childhood obesity is associated inversely with socioeconomic status (SES).^11,^^12^ In contrast, wealthier children in most developing countries are at a greater risk of obesity.^13^ This trend is referred to as the ‘social drift’ phenomenon.^10^ The main sociodemographic factors known to be associated with body weight regulation (including malnutrition as well as overweight and obesity) in children include gender, age, race, parents’ education levels and occupation, household size, residential density and geographical region.^14^ The need to assess factors that impact on the nutritional status of South African adolescents of all ethnicities and social backgrounds is necessary so that effective intervention programmes can be implemented at an early stage.^15^

Few studies in South Africa have investigated the impact of sociodemographic factors on the nutritional status of adolescents.^3^^,16^ The aim of this article is to report on the nutritional status of children aged 13–15 years and to indicate the association between the children's nutritional status and their sociodemographic characteristics.

### Significance of the study

Studies on the prevalence of malnutrition and overweight and obesity have always focused on children, particularly under the age of five years. There is, however, a steady increase in the investigation of the nutritional status of adolescents and factors contributing to their being either malnourished or overweight. The current study aims to make a contribution to this body of evidence in a South African setting.

## Research methods and design

### Population and setting

The study population comprised learners aged 13–15  years old (*N* = 640) who attended public comprehensive secondary schools in Bloemfontein and represented all the socioeconomic groups. A cross-sectional study of learners from 26 public secondary schools in Bloemfontein was performed.

### Data collection methods

Approval to conduct the study was obtained from the Free State Department of Health. Thereafter, the school principals were contacted and the aim of the study was explained to them. The sample size of 640 children was determined based on relevant literature as well as the amount of time allocated by the school principals compared to the number of interviewers (five, including the principal researcher) available to conduct the research. A proportionally representative number of children to participate in the study was determined using quota sampling for each school. Alphabetical lists of all learners aged 13–15 years were then obtained from the schools’ principals and a random sample of children was selected from these lists.

Before the study commenced, four fieldworkers were trained on standardised anthropometric methods, interviewing of children and completion of the questionnaire. A pilot study was carried out before commencement of the main study in order to test the questionnaire. Ten children from one of the schools were interviewed and measured and, where necessary, adaptations and changes were made to the questionnaire. These children were not included in the final sample.

A research room was identified at each school and the researcher and field workers used structured interviews to obtain sociodemographic data from the learners. The interviews were conducted in the children's language of choice – English, Afrikaans, Sesotho or Setswana. The structured sociodemographic questionnaire included information on gender, language, household composition, type and size of dwelling, available cooking and storage facilities, as well as information on the main economic contributor of the household with regard to level of education and occupation. The main contributors were defined as individuals in the household who contributed the most income and were classified as specialist professionals (e.g. doctors, professors, company directors), graduate professionals (e.g. teachers, nurses, managers), skilled individuals (clerks, secretaries, retail workers) or unskilled individuals (cleaners, domestic workers, gardeners). Based on the occupation and highest education level of the main contributor to the household, the children were classified as having a high, middle or low SES.

Standardised methods and techniques^17^ were used by the researcher and field workers for anthropometric measurements. Weight was measured on a Seca beam balance to the nearest 0.1 kg with the children wearing minimal clothing and no shoes. Height was measured using a stadiometer (Seca 214) to the nearest 0.5 cm.^17^

The children's body mass index (BMI)-for-age *z*-scores (BAZ) and height-for-age *z*-scores (HAZ) were generated using the World Health Organization's (WHO) Anthro 2005 program, Beta version.^18^ Nutritional status was classified as follows:^18^

- underweight/stunted if BAZ and HAZ, respectively, were less than two standard deviations below the median reference.
- at risk of underweight/stunting if BAZ and HAZ, respectively, were less than one but more than two standard deviations below the median reference.
- at risk of overweight if BAZ was more than one but less than two standard deviations above the median reference.
- overweight if BAZ was more than two but less than three standard deviations above the median.
- obese if BAZ was more than three standard deviations above the median.

Waist circumference tends to be a better predictor of risk for metabolic disease than BMI.^19^ Ethnic-specific percentiles of waist circumference-for-age and gender were used to classify children according to waist circumference-associated risk. Waist circumferences ≥ 75th and ≥ 90th percentiles indicated a high and a very high risk for comorbidities, respectively.^20^

To ensure reliability, 10% of the sampled children were re-interviewed by the researcher two weeks after the initial interview. Anthropometric measurements were obtained in triplicate on the same day in order to ensure reliability and accuracy. Where it was found that an answer to a question differed by more than 10% between the first and second interview, the question was considered unreliable.

### Statistical analysis

Descriptive statistics, namely medians and percentiles for continuous data and frequencies and percentages for categorical data, were calculated per group. Differences between groups were compared by means of *p*-values using the chi-square test. Stepwise logistic regression analysis was performed and the odds ratios, 95% CI and *p*-values were calculated for each risk factor in the final model. A *p-*value of < 0.05 was considered to be of statistical significance.

### Ethical considerations

The study was approved by the Ethics Committee of the Faculty of Health Sciences, University of the Free State (ETOVS NR. 245/05). The Free State Department of Education and the schools’ principals gave permission for the study to be conducted in schools. The learners’ parents/guardians had to give written consent for the learners to participate in the study and the learners themselves had to give written assent to indicate their willingness to participate. Confidentiality and anonymity were ensured throughout the duration of the study.

## Results

A response rate of 67% was achieved, with 415 children (175 boys and 240 girls) giving consent to participate in the study.

### Sociodemographic data

Results of the participants’ sociodemographic data are presented in Table 1. Most of the children were black (*n* = 339; 81.7%), followed by white children (*n* = 52; 12.5%). The children's median age was 14.3 years and most were in grade 8 (*n* = 363; 87.5%).

**TABLE 1:** Sociodemographic variables of children 13–15 years of age.

Variable	Boys *n* (%)	Girls *n* (%)	Total *n* (%)
**School grade**			
Grade 8	160 (91.4)	203 (84.6)	363 (87.5)
Grade 9	15 (18.6)	37 (15.4)	52 (12.5)
**Race**			
Black	140 (80)	199 (82.9)	339 (81.7)
Coloured	8 (4.6)	13 (5.4)	21 (5.1)
Indian	0 (0)	1 (0.4)	1 (0.24)
White	26 (14.9)	26 (10.8)	52 (12.5)
Other (Chinese)	1 (0.6)	1 (0.4)	2 (0.48)
**Home language**			
Afrikaans	33 (18.9)	31 (12.9)	64 (15.4)
English	5 (2.9)	10 (4.2))	15 (3.6)
Sesotho	72 (41.1)	99 (41.3)	171 (41.2)
Setswana	38 (21.7)	63 (26.3)	101 (24.3)
isiXhosa	26 (14.9)	35 (14.6)	61 (14.7)
Chinese	1 (0.6)	2 (0.8)	3 (0.72)
**Type of dwelling**			
Brick or concrete	158 (90.3)	214 (89.2)	372 (89.7)
Traditional mud	2 (1.1)	0 (0)	2 (0.48)
Corrugated iron	13 (7.4)	19 (7.9)	32 (7.7)
Apartment	2 (1.1)	6 (2.5)	8 (1.9)
Wood	0 (0)	1 (0.4)	1 (0.24)
**Type of toilet**			
Flush	154 (88)	209 (87.1)	363 (87.5)
Pit	4 (2.3)	8 (3.3)	12 (2.9)
Bucket	16 (9.1)	20 (8.3)	36 (8.7)
Mobile toilet	1 (0.6)	3 (1.3)	4 (0.96)
**Fuel mostly used for cooking**			
Electric	158 (90.3)	217 (90.4)	375 (90.4)
Gas	6 (3.4)	4 (1.7)	10 (2.4)
Paraffin	9 (5.1)	19 (7.9)	28 (6.8)
Wood, coal	2 (1.1)	-	2 (0.48)
**Working refrigerator in household**			
Yes	159 (90.9)	209 (87.1)	368 (88.7)
No	16 (9.1)	31 (12.9)	47 (11.3)
**Working stove in household**			
Yes	164 (93.7)	222 (92.5)	386 (93.0)
No	11 (6.3)	18 (7.5)	29 (6.99)
**Working microwave oven in household**			
Yes	118 (67.4)	143 (59.6)	261 (62.9)
No	57 (32.6)	97 (40.4)	154 (37.1)
**Working radio and/or television in household**			
Yes	173 (98.9)	236 (98.3)	409 (98.6)
No	2 (1.1)	4 (1.7)	6 (1.4)
**Main contributor to the household**			
Mother	66 (37.7)	86 (35.8)	152 (36.3)
Father	82 (46.9)	110 (45.8)	192 (46.3)
Sister/brother	9 (5.14)	12 (5.0)	21 (5.1)
Grandparent	9 (5.14)	20 (8.3)	29 (6.99)
Uncle/aunt	9 (5.14)	12 (5.0)	21 (5.1)
**Occupation of main contributor of income in household**			
Unskilled	48 (27.4)	87 (36.3)	135 (32.5)
Skilled	69 (39.4)	82 (34.2)	151 (36.4)
Graduate	48 (27.4)	63 (26.2)	111 (26.8)
Specialist	10 (5.8)	8 (3.3)	18 (4.3)
**Main contributor's highest level of education**			
Primary	12 (6.9)	35 (14.6)	129 (31.1)
High school	48 (27.4)	7 (30.0)	119 (28.7)
Matric	53 (30.3)	66 (27.5)	120 (28.9)
Post-matric	62 (35.4)	67 (27.9)	47 (11.3)
**Socioeconomic status category**			
Low	32 (18.3)	74 (30.8)	106 (25.5)
Medium	95 (54.3)	109 (45.4)	204 (49.2)
High	48 (27.4)	57 (23.8)	105 (25.3)

Most children stayed in brick houses (*n* = 372; 89.7%) and electricity (*n* = 374; 90.4%) was used as the main type of fuel for cooking in most households. Household appliances, including a working stove (gas, coal or electricity: *n* = 386; 93.0%) and a working refrigerator (*n* = 367; 88.7%), were available in most of the households.

For approximately half of the children (*n* = 192; 46.3%), the father was indicated as the one contributing most income to the household, followed by the mother (*n* = 152; 36.3%). Most of the main contributors to the household were skilled workers (*n* = 151; 36.4%) (clerks, office assistants, sales persons), followed by unskilled workers (*n* = 135; 32.5%) (domestic workers, cleaners, gardeners and contract workers). Only 47 (11.3%) of the children's parents who were contributors had post-matric education. Based on the parent's highest level of education and occupation, 204 (49.2%) of the children were categorised to be of medium socioeconomic status.

Table 2 shows the results of the children's anthropometric measurements. Based on BMI-for-age, 244 (56.7%) of the children could be categorised as having a normal weight. Significantly more girls (*n* = 18; 7.5%) were overweight and obese than boys (*n* = 7; 4.0%) (*p* = 0.0002), whilst on the other hand, significantly more boys (*n* = 40; 23.0%) were wasted than girls (*n* = 24; 10.0%) (*p* = 0.0003). Similar to BMI-for-age, most of the children had a normal height-for-age (*n* = 273; 65.7%). Another significant difference between genders was that 18 (10.3%) of the boys were more severely stunted, compared to 10 (4.2%) of the girls (*p* = 0.01).

**TABLE 2:** Anthropometric data of children 13–15 years of age.

Variable	Boys *n* (%)	Girls *n* (%)	Total *n* (%)
**Body mass index-for-age z score category (BAZ)**	***n* = 174**	***n* = 240**	***n* = 414**
Severely wasted	8 (4.6)	6 (2.5)	14 (3.4)
Wasted	40 (23.0)*	24 (10)	64 (14.9)
Normal weight	103 (59.2)	141 (58.8)	244 (56.7)
At risk of overweight	16 (9.2)	51 (21.3)	67 (15.6)
Obese	7 (4.0)	18 (7.5)	25 (6.0)
Overweight and obese	23 (13.2)	69 (28.8)*	92 (21.6)
**Height-for-age z score category (HAZ)**	***n* = 59**	***n* = 83**	***n* = 142**
Severely stunted	18 (10.3)*	10 (4.2)	28 (6.8)
Stunted	41 (23.6)	73 (30.4)	114 (27.5)
Normal	115 (66.1)	157 (65.4)	273 (65.7)
**Waist circumference (percentile)#**	***n* = 175**	***n* = 240**	***n* = 415**
< 10	32 (18.3)	29 (12.1)	61 (14.7)
10–24.9	43 (24.6)	35 (14.6)	78 (18.8)
25–49.9	43 (24.6)	81 (33.8)	124 (29.9)
50–74.9	35 (20.0)	51 (21.3)	86 (20.7)
75–89.9	18 (10.3)	35 (14.6)	53 (12.8)
≥ 90	4 (2.3)	9 (3.8)	13 (3.1)

*, Statistically significant difference;

^#^, Percentiles based on American cut-off points.^17^

Waist circumference was also normal in most of the children, with only 68 (15.9%) of the children being above the 75th percentile of the ethnic-specific cut-off points. Although not statistically significant, more girls (*n* = 44; 18.4%) than boys (*n* = 22; 12.6%) had a waist circumference above the 75th percentile.

Table 3 shows the association between BMI-for-age and height-for-age with regard to different sociodemographic and economic indices. Because of the under-representation of other racial groups, associations between BMI and height-for-age could not be established with regard to race and home language.

**TABLE 3:** Body mass index-for-age and height-for-age comparisons according to socioeconomic indicators of children 13–15 years of age.

Demographics	Body mass index-for-age (BAZ)					Height-for-age (HAZ)	
	Underweight *n* (%)	At risk *n* (%)	Normal weight *n* (%)	At risk *n* (%)	Overweight/ obese *n* (%)	Stunted *n* (%)	At risk *n* (%)
**Race**							
Black	13 (3.9)	53 (15.7)	199 (58.9)	52 (15.4)	21 (6.2)	27 (8.0)	103 (30.5)
Coloured	1 (4.8)	1 (4.8)	16 (76.2)	3 (14.3)	0 (0)	1 (4.8)	6 (28.6)
Indian	0 (0)	0 (0)	1 (100)	0 (0)	0 (0)	0 (0)	0 (0)
White	0 (0)	10 (19.2)	26 (50.0)	12 (23.1)	4 (7.7)	0 (0)	5 (9.6)
Other (Chinese)	0 (0)	0 (0)	2 (100)	0 (0)	0 (0)	0 (0)	0 (0)
**Home language**							
Afrikaans	1 (1.6)	9 (14.1)	40 (62.5)	10 (15.6)	4 (6.3)	1 (1.6)	11 (17.2)
English	0 (0)	3 (20.0)	7 (46.7)	5 (33.3)	0 (0)	0 (0)	2 (13.3)
Sesotho	2 (1.2)	22 (12.9)	106 (62.0)	30 (17.5)	11 (6.4)	18 (10.5)	53 (31.0)
Setswana	8 (8.0)	24 (24.0)	45 (45.0)	18 (18.0)	5 (5.0)	8 (8.0)	34 (34.0)
isiXhosa	3 (4.9)	5 (8.2)	44 (72.1)	4 (6.6)	5 (8.2)	1 (1.6)	14 (23.0)
Chinese	0 (0)	1 (33.3)	2 (66.7)	0 (0)	0 (0)	0 (0)	0 (0)
**Parents’ occupation**							
Unskilled	5 (3.7)	19 (14.2)	79 (58.9)	21 (15.7)	10 (7.5)	12 (9.0)*	41 (30.6)
Skilled	7 (4.6)	31 (20.5)*	89 (58.9)	18 (11.9)	6 (4.0)	13 (8.6)	45 (29.8)
Graduate	2 (1.8)	10 (9.0)	65 (58.6)	25 (22.5)	9 (8.1)*	3 (2.7)	23 (20.7)
Specialist	0 (0)	4 (22.2)	11 (61.1)	3 (16.7)	0 (0)	0 (0)	5 (27.8)
**Parents’ level of education**							
Post-Matric	2 (4.3)	4 (8.5)	29 (61.7)	6 (12.8)	6 (12.8)	3 (6.4)	14 (29.8)
Matric	6 (5.0)	20 (16.8)*	71 (59.7)	16 (13.5)	6 (5.0)	12 (10.1)*	44 (36.9)
High school	5 (4.2)	24 (20.2)	65 (54.6)	20 (16.8)	5 (4.2)	8 (6.7)	30 (25.2)
Primary school	1 (0.8)	16 (12.4)	79 (61.2)	25 (19.4)	8 (6.2)	5 (3.9)	26 (20.2)
**Socioeconomic status**							
Low	5 (4.8)	11 (10.5)*	65 (61.9)	16 (15.2)	8 (7.6)	9 (8.6)	33 (31.4)*
Medium	8 (3.9)	42 (20.6)	113 (55.4)	32 (15.7)	9 (4.4)	16 (7.8)	62 (30.4)*
High	1 (0.9)	11 (10.5)*	66 (62.9)	19 (18.1)	8 (7.6)	3 (2.9)	19 (18.1)

*, Statistically significant difference

The prevalence of wasting was significantly higher in children of skilled parents than those of graduate parents (*n* = 31; 20.5% and *n* = 10; 9.0%, respectively) (*p* = 0.01). Significantly more children of graduate parents (*n* = 9; 8.1%) were overweight/obese than those of skilled parents (*n* = 6; 4.0%) (*p* = 0.004). Considering overall SES based on parents’ level of education and parents’ occupation, children classified as being of medium SES (*n* = 42; 20.6%) were significantly more wasted than those of low (*n* = 11; 10.5%) and high SES (*n* = 11; 10.5%) (*p* = 0.03). A significant association could not be made between overall SES and overweight/obesity in these children.

Only five children of parents with specialist occupations and three of those with parents with graduate occupations were either stunted or severely stunted. The difference in the prevalence of severe stunting was significant between unskilled (*n* = 12; 9.0%) and graduate (*n* = 3; 2.7%) parents’ children (*p* = 0.03). Despite the fact that there was no significant association between weight and parental level of education, the children whose parents had matric as their highest level of education (*n* = 12; 10.1%) were significantly more severely stunted than those whose parents had primary school (*n* = 5; 3.9%) as their highest level of education (*p* = 0.003). Furthermore, children of low SES (*n* = 33; 31.4%) and medium SES (*n* = 62; 30.4%) were significantly more stunted than children of high SES (*n* = 19; 18.1%) (*p* = 0.03 and 0.02, respectively).

On logistic regression (Table 4), the odds of being underweight were higher in boys and less likely in children with a medium to high SES. Children whose parents had a level of education of high school, matric and post-matric were more likely to be overweight. Additionally, children whose parents had graduate or specialist occupations were less likely to be stunted.

**TABLE 4:** Logistic regression predicting nutritional status in children 13–15 years of age.

Variable	Indicator	Odds ratio	95% CI	*p*-value
Parental education (high school and upwards)	Overweight	0.373	0.148–1.071	0.0397*
Male gender	Underweight	2.776	1.673–4.674	0.0001*
High socioeconomic status	Underweight	0.475	0.252–0.852	0.0145*
Graduate and specialist occupations	Stunting	0.496	0.307–0.786	0.0031*

*, Statistically significant

## Discussion

The current study was the first of its kind to be carried out in the Free State Province of South Africa to investigate factors associated with adolescents’ nutritional status. The study was conducted in Bloemfontein, an urban area of the province consisting largely of built-up areas and informal settlements. Consequently, most of the children lived in brick houses (89.7%) and had access to water and sanitation facilities (87.5%). McVeigh, Norris and DeWet^21^ obtained similar results with their sample of children living in Johannesburg, South Africa. In their study, 88% of the children lived in brick houses, 95% and 94% of these households had a television set and refrigerator, respectively, whilst only 39% owned a microwave.^21^ The fact that our study was based in an urban area also meant that a high percentage of the children had storage and cooking facilities, such as fridges, stoves and microwave ovens, available in their homes.

Using the WHO classification for BMI-for-age, the prevalence of overweight and obesity (> + 2 SD) was relatively low (6%). However, the number of children classified as being at risk for overweight was higher (15.6%). The coexistence of undernutrition in these children, as indicated by the high number of wasted (< -2 SD) and severely wasted children (< -3 SD) (18.3% combined), confirms the double burden of over- and undernutrition common in developing countries.^13,22^ Almost a third (27.5%) of the children in this study were stunted, whilst 6% were severely stunted. These figures emphasise the fact that even though there is an observed increase in the prevalence of overweight and obesity, the problem of undernutrition continues to affect South African children.^23^ The high prevalence of wasting and stunting can also be seen as a reflection of the high levels of underdevelopment typical of the Free State Province^16,24^ and food poverty experienced by most South African families.^25^

Similar to other South African studies,^16,24,26^ the prevalence of overweight and obesity in our study was higher amongst girls than boys, even though these studies used different methods of interpreting nutrition status. Furthermore, as confirmed by logistic regression, wasting and stunting occurred more amongst boys than girls. These differences in nutritional status as a result of gender can possibly be ascribed to earlier sexual maturation in girls, which influences body fat^26,27^ or possibly to the fact that the boys in this study might have been physically more active than the girls, as reported elsewhere.^28^

The association between waist circumference and BMI in our study was statistically significant. Only a small percentage of the children (15.9%) had a waist circumference above the 75th percentile, compared with 27.9% of the children in a Mexican study.^29^ In children where waist circumference was above the suggested cut-off point, the risk of metabolic complications is increased. Adolescents with a waist circumference above the 90th percentile have been shown to have higher concentrations of low density lipoprotein (LDL) cholesterol, triglycerides and insulin, as well as lower concentrations of high density lipoprotein (HDL),^20,^^30^ increasing their risk of developing chronic diseases of lifestyle in later years.

SES has been established as a predictor of nutritional status in several studies.^13,26,^^31^ Pathways through which SES may be associated with nutritional status include income, education and occupation.^3^ In developed countries, lower SES has been found to be a strong predictor of obesity,^13,32^ whilst a high SES status in developing countries is associated with an increased risk for obesity.^31,33^ The current study failed to find any significant linear association between the prevalence of obesity and overall SES.

Significantly more children whose parents had graduate occupations were overweight/obese and tended to be less wasted than children whose parents were skilled labourers. The THUSA BANA study,^16^ conducted in the North-West Province of South Africa, also compared children's weight status with their parents’ occupation. They found that children whose parents were employed as domestic or contract workers (unskilled) were less inclined to be overweight/obese than children whose parents had professional or business occupations (graduate and specialists).^16^ This could possibly be attributed to more money being available to spend on food in these households than in the domestic or contract workers’ households. In contrast to this current study, Ekelund et al.^34^ established an inverse association between the parents’ education level and BMI in the girls in their study in Stockholm.

Whilst no associations could be established with overweight and obesity and SES, stunting and underweight presented a different picture. The odds of being underweight were low in children with a medium to high SES and the odds of being stunted were low in children whose parents had graduate or specialist occupations. Stunting in the children was associated with being from a low SES group, having a parent who had completed secondary education and having a parent who had an occupation categorised as unskilled. These results confirm the fact that household food insecurity, low parental education and low levels of employment are amongst the main determinants of stunted growth.^35^ Education and employment typically translate into opportunities to earn money and therefore secure adequate food for the family. Furthermore, a low level of education may predict a deficiency in nutritional knowledge and thereby affect children's nutritional status.^3^

Although race has been noted as a predictor of nutritional status in some studies, no such association could be made in our study, mainly because of the under-representation of race groups other than black people (81.7%) and white people (12.5%). The THUSA BANA study found that the prevalence of obesity was twice as high in white children compared with the other racial groups.^16^ Mamabolo et al.^36^ also reported that white primary school children had a higher body weight than their black counterparts. Nationally, Indian children (25.3%) were more overweight than the white (23.4%) and black (16.6%) children.^24^ It is important to note, however, that in all these studies, a limited number of white children were included and the data may not be representative of the total population.

## Recommendations

Policies on the prevention of under- and overnutrition in children and adolescents need to be planned and supported in order to address the double burden of malnutrition in South Africa.

Because of the ambiguity relating to sociodemographic factors and their effect on nutritional status, researchers and/or programme planners need to conduct in-depth assessments of possible factors leading to under- or overnutrition in their targeted intervention communities prior to commencement of the programme.

## Conclusion

The effect of sociodemographic factors on the prevalence of under- and overnutrition remains important when effective intervention programmes have to be planned. However, limited literature is available on sociodemographic factors in South Africa and its influence on adolescent nutritional status. This study aimed to contribute to the literature by focusing on the Free State Province in South Africa, which is somewhat neglected with regard to research in this specific area. A coexistence of under- and overnutrition was found in this study population, which is typical of developing countries. Male gender and the parents’ occupation and level of education were indicated as determinants of nutritional status.
